# Cooperative effects of oocytes and estrogen on the forkhead box L2 expression in mural granulosa cells in mice

**DOI:** 10.1038/s41598-022-24680-x

**Published:** 2022-11-23

**Authors:** Haruka Ito, Chihiro Emori, Mei Kobayashi, Natsumi Maruyama, Wataru Fujii, Kunihiko Naito, Koji Sugiura

**Affiliations:** 1 Laboratory of Applied Genetics, Department of Animal Resource Sciences, Graduate School of Agricultural and Life Sciences, The University of Tokyo, Tokyo, Japan; 2 Present Address: Department of Experimental Genome Research, Research Institute for Microbial Diseases, Osaka University, Suita, Osaka, Japan

**Keywords:** Developmental biology, Endocrinology

## Abstract

Forkhead box L2 (FOXL2) plays a critical role in the development and function of mammalian ovaries. In fact, the causative effects of FOXL2 misregulations have been identified in many ovarian diseases, such as primary ovarian insufficiency and granulosa cell tumor; however, the mechanism by which FOXL2 expression is regulated is not well studied. Here, we showed that FOXL2 expression in ovarian mural granulosa cells (MGCs) requires stimulation by both oocyte-derived signals and estrogen in mice. In the absence of oocytes or estrogen, expression of FOXL2 and its transcriptional targets, *Cyp19a1* and *Fst* mRNA, in MGCs were significantly decreased. Moreover, expression levels of *Sox9* mRNA, but not SOX9 protein, were significantly increased in the FOXL2-reduced MGCs. FOXL2 expression in MGCs was maintained with either oocytes or recombinant proteins of oocyte-derived paracrine factors, BMP15 and GDF9, together with estrogen, and this oocyte effect was abrogated with an ALK5 inhibitor, SB431542. In addition, the FOXL2 level was significantly decreased in MGCs isolated from *Bmp15*^−/−^ /*Gdf9*^+/−^ mice. Therefore, oocyte, probably with estrogen, plays a critical role in the regulation of FOXL2 expression in mural granulosa cells in mice.

## Introduction

The development of ovarian antral follicles accompanies the formation of the antral cavity, which divides the granulosa cells of preantral follicles into two functionally distinct populations: cumulus cells and mural granulosa cells (MGCs). Cumulus cells surround the oocyte and are specialized to support its development, while MGCs line the follicular wall and mainly have endocrine functions. The coordinated development of these granulosa cell populations is vital for normal female fertility, as a deficiency in either cell type would result in defects in the production of functional oocytes or ovarian hormone production.

While cumulus cells and MGCs both originate from preantral granulosa cells, the mechanism governing the differentiation of these cell types is not fully resolved. Follicle stimulating hormone (FSH) is clearly one of the critical determinants of this process, and the development of the MGC phenotype depends, to a great extent, on the FSH stimulation. In fact, the expression of many transcripts that are more enriched in MGCs than in cumulus cells, such as *Lhcgr* encoding luteinizing hormone (LH) receptor, depends upon FSH stimulation^1^. On the other hand, the development of a cumulus cell phenotype depends on factors secreted from oocytes. Growth differentiation factor 9 (GDF9) and bone morphogenetic protein 15 (BMP15) are members of the transforming growth factor β (TGF-β) superfamily produced by oocytes. *Gdf9*-deficient female mice are infertile because of the arrest of follicle development at the primary follicle stage^2^. Whereas *Bmp15* null females are subfertile, the additional deletion of a single *Gdf9* allele (i.e., *Bmp15*^-/-^/*Gdf9*^+/-^; hereafter double-mutant, DM) results in female infertility at least in part due to the defective development of cumulus cells^3^. Therefore, the FSH from outside the follicles, and the oocyte-derived signal from within the follicles establish the opposing signal gradient which is crucial for the specification of the cumulus cell and MGC phenotypes^1^.

Evidence indicates that oocytes are required for the development and maintenance of not only cumulus cells, but also MGCs. The profound effects of the oocyte-derived signal on MGC development were well illustrated by a previously reported experiment in which mid-growth-stage oocytes from preantral follicles were recombined with follicular somatic cells from newborn ovaries^4^. In the re-aggregated ovary, the rate of development of both cumulus cells and MGCs was accelerated by the mid-growth stage oocytes. Moreover, removing the cumulus-oocyte complex (COC) from follicles in vivo results in precocious luteinization of MGCs in rabbits^5^, and similarly, oocytes suppress luteinization of rat MGCs in vitro^6^. These observations indicate that oocyte-derived signals are critical not only for MGC development, but also for maintaining the MGC characteristics/phenotype; however, the mechanism by which oocytes influence these processes is ill-defined.

In addition to oocytes and FSH, follicle development requires signals of estrogen. Mice deficient in the *Esr2* gene encoding estrogen receptor 2 (also known as estrogen receptor-β), expressed in all types of granulosa cells, exhibit abnormal hormonal responsiveness and lower ovulation rate^7^. Moreover, loss of both *Esr1* (encoding estrogen receptor-α) and *Esr2* results in infertility due to defects in the differentiation of granulosa cells, and seminiferous tubule-like tissue is formed in the ovaries of the aged mutant females^8^. In vitro analyses also revealed that estrogen and oocyte signals coordinately affect the development and functions of cumulus cells^9,10^. Therefore, the estrogen signal is critical for the development and function of both types of granulosa cell.

Forkhead box L2 (FOXL2) is a well-known transcription factor essential for female sex determination as well as for the normal development and function of mammalian ovaries. In *Foxl2*-deficient ovaries, granulosa cells fail to proceed to the squamous-to-cuboidal transition that is normally observed during the transition of primordial to primary follicles^11,12^. In contrast, deletion of *Foxl2* in adult ovaries results in the elevated expression of testis-specific transcripts, including SRY-box9 (SOX9), in MGCs, and ultimately in the trans-differentiation into Sertoli-like cells^13^. These observations indicate that FOXL2 is indispensable for the normal development and maintenance of granulosa cells, especially for MGCs. In fact, our recent in silico analysis of a transcriptomic comparison between cumulus cells and MGCs identified FOXL2 as a critical transcriptional regulator responsible for differentiation of the MGC lineage^14^. FOXL2 is expressed at higher levels in MGCs, whereas cumulus cells express lower levels due to the suppression of FOXL2 expression by oocytes. As mentioned above, although the MGC phenotype depends to a great extent on FSH stimulation, the FOXL2 levels in MGCs were not maintained in vitro even when the cells were stimulated with FSH^14^. This suggests that some unknown factors present within follicles, other than FSH, are responsible for the maintenance of FOXL2 expression, and thus for the maintenance of MGC characteristics/phenotypes.

Therefore, the present study was conducted to unveil the mechanism for maintaining FOXL2 expression in MGCs. Since both the oocyte and the estrogen signals are critical for regulating MGC development, we tested the possibility that these signals are responsible for the maintenance of FOXL2 expression in MGCs.

## Results

### MGCs lose FOXL2 expression in vitro

**Figure 1:**
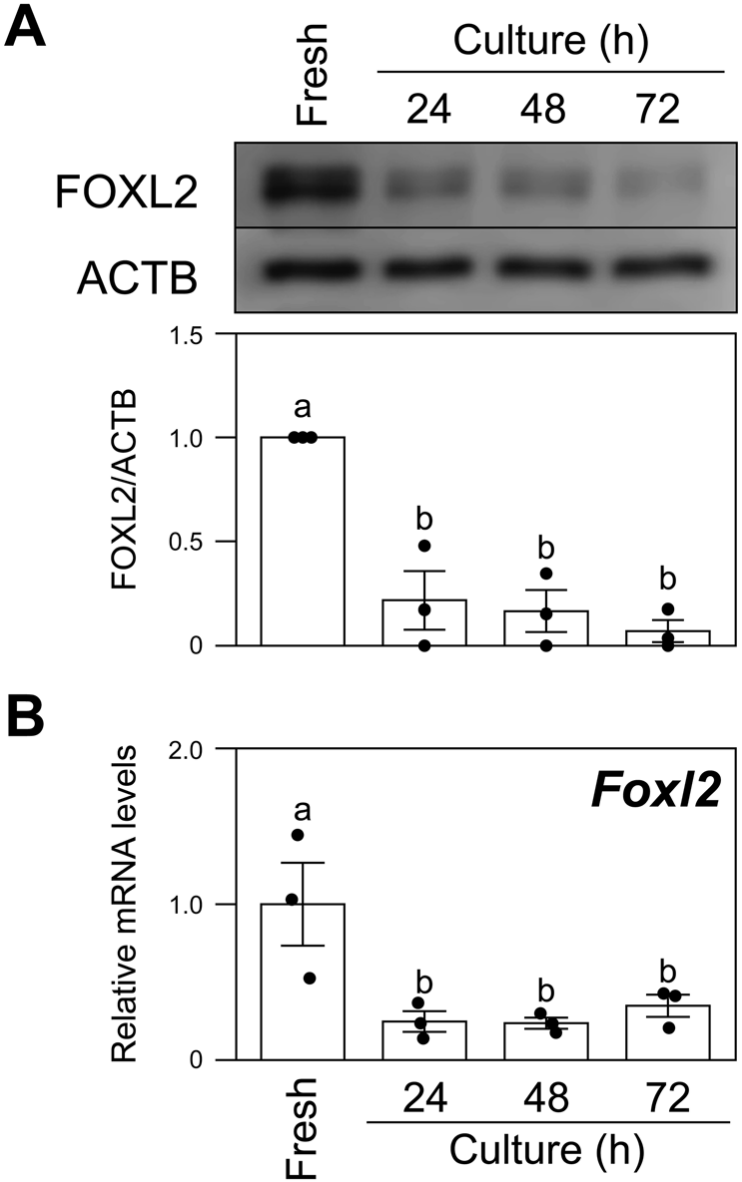
Effects of culture on the expression kinetics of FOXL2 in MGCs. Freshly isolated MGCs (Fresh) and MGCs cultured for 24, 48, and 72 h were examined. (**A**) The expression levels of FOXL2 protein were examined by western blotting (upper panel). The relative intensities of protein bands were quantified (lower panel) (n = 3). (**B**) The expression levels of *Foxl2* mRNA were examined by qPCR (n = 3). Different letters (a and b) denote significant differences (p < 0.05).

Previously, we reported that FOXL2 expression in MGCs was not maintained in culture^14^. To assess the kinetics of FOXL2 expression in cultured MGCs in more detail, we here examined the expression levels of the FOXL2 protein and *Foxl2* mRNA (Fig. 1A,B). While freshly isolated MGCs exhibited a high expression level of FOXL2 protein, the levels were remarkably decreased after 24 h of culture. The FOXL2 protein expression in MGCs became barely detectable after 72 h of culture (Fig. 1A). Similarly, the *Foxl2* mRNA levels declined significantly compared with those in freshly isolated MGCs during the culture. These results suggest that the elevated FOXL2 expression in MGCs requires a factor that does not exist (or multiple factors that do not exist) under the present culture condition.

**Figure 2:**
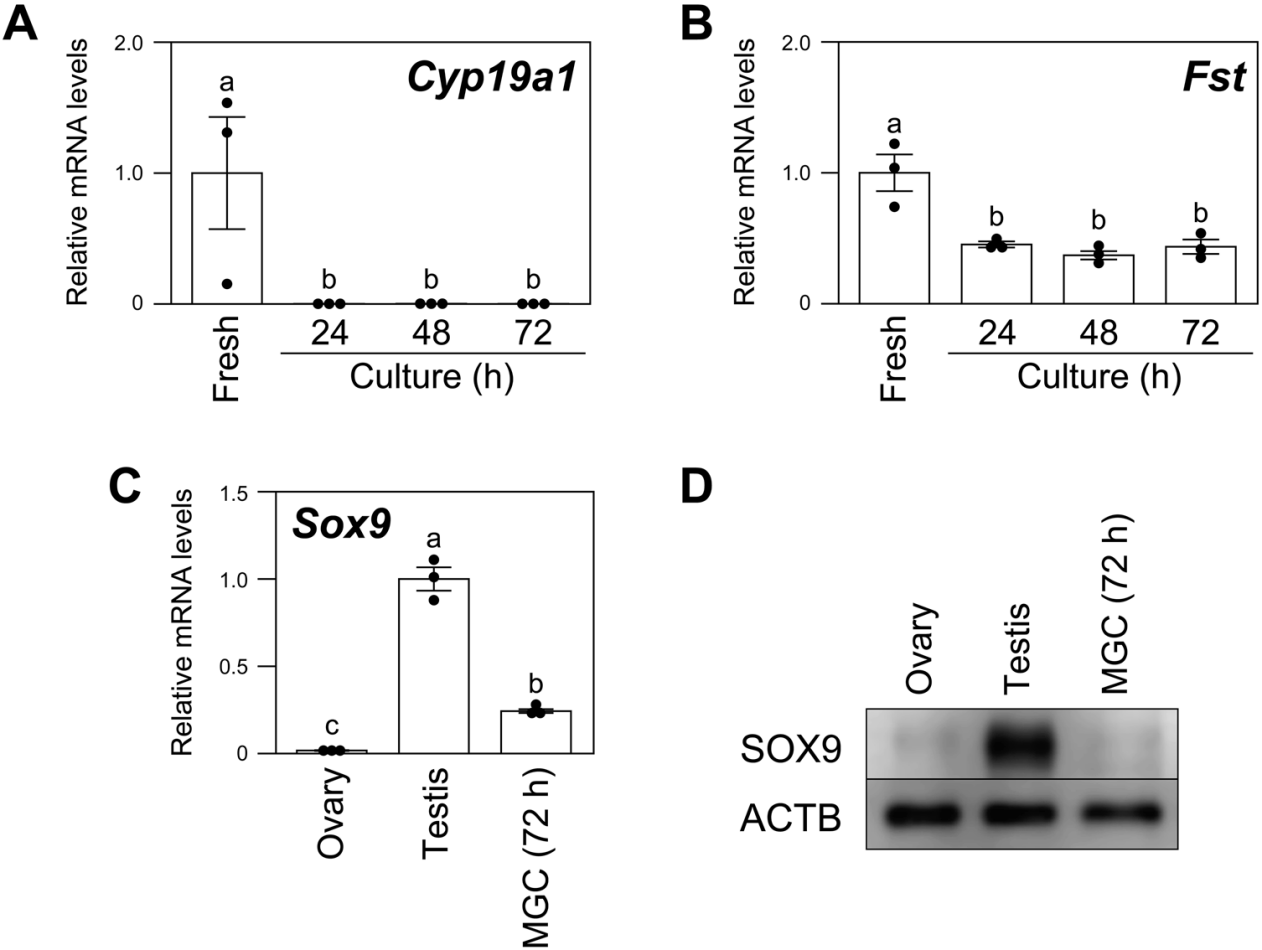
Effects of culture on the expression levels of transcriptional targets of FOXL2 in MGCs. Freshly isolated MGCs (Fresh) and MGCs cultured for 24, 48, and 72 h were examined for the expression kinetics of (**A**) *Cyp19a1*, (**B**) *Fst*, and (**C**) *Sox9* mRNAs by qPCR (n = 3). (**D**) The expression of SOX9 protein was examined by western blotting. Different letters (a, b, and c) denote significant differences (*p* < 0.05).

CYP19A1 (also known as aromatase) and FST (follistatin) are important regulators of the development and function of mammalian ovaries^15,16^ and are known to be transcriptional targets of FOXL2^17–19^*.* Therefore, the effects of the culture on the expression levels of these transcriptional targets of FOXL2 were examined (Fig. 2). qPCR analysis revealed that the expression levels of both *Cyp19a1* and *Fst* transcripts in MGCs were significantly decreased during the culture (Fig. 2A,B). Therefore, the decline in FOXL2 levels during the culture appears to be functionally significant.

The *Foxl2* deletion in ovaries of adult mice results in trans-differentiation of granulosa cells into Sertoli-like cells with elevated expression of a Sertoli cell marker, SOX9^13^. Therefore, we next examined the SOX9 expression in the cultured (FOXL2-decreased) granulosa cells. As shown in Fig. 2C, the expression level of *Sox9* mRNA was significantly increased in the monolayer-cultured MGCs compared with ovary; however, the expression level was still significantly lower than in the testis. In addition, SOX9 protein expression was not detectable in the MGCs up to at least 72 h of culture (Fig. 2D).

Taken together, these results suggest that the high level of FOXL2 expression in MGCs is not regulated cell autonomously, but rather requires some factor(s) present in ovarian follicles.

### Oocyte-derived signals and E2 promote FOXL2 expression in MGCs in vitro

**Figure 3:**
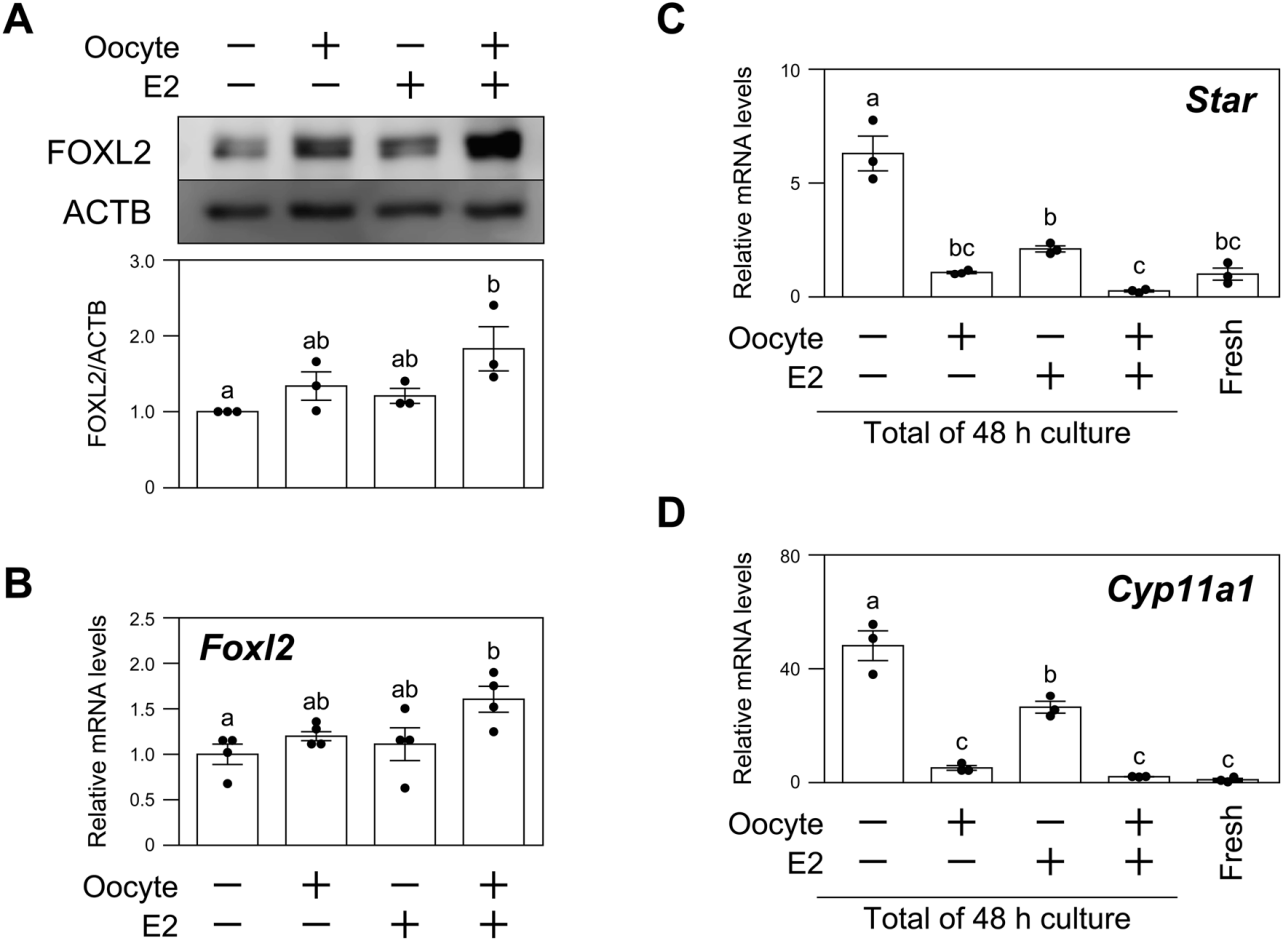
Effects of oocyte-derived signals and 17β-estradiol on the expression of FOXL2 in MGCs in vitro. MGCs were cultured with or without oocytes (2 oocytes/μl) and/or 17β-estradiol (E2) for 24 h. (**A**) The expression levels of FOXL2 protein were examined by western blotting (upper panel). The relative intensities of protein bands were quantified (lower panel) (n = 3). (**B**) The expression levels of *Foxl2* mRNA were examined by qPCR (n = 4). The expression levels of (**C**) *Star* and (**D**) *Cyp11a1* mRNAs were examined by qPCR (n = 3). Different letters (a, b and c) denote significant differences (*p* < 0.05).

Oocyte-derived signals and estrogen cooperatively play an important role in the development of granulosa cells in mice^9,10^. Therefore, we hypothesized that both oocyte-derived signals and estrogen may influence FOXL2 expression in MGCs, and therefore, we next tested this possibility (Fig. 3A,B). While oocyte-co-culture or 17β-estradiol (E2)-supplementation exhibited only a slight effect on the FOXL2 protein level in MGCs, co-treatment with oocytes and E2 significantly promoted FOXL2 expression in MGCs (Fig. 3A). Likewise, *Foxl2* mRNA level was significantly increased by treatment with both oocytes and E2 (Fig. 3B).

To analyze whether the spontaneous luteinization of MGCs contributes to the FOXL2 expression kinetics in our culture system, we quantified the mRNA expressions of *Star* and *Cyp11a1* in the cultured MGCs (Fig. 3C,D). We found that the expressions of *Star* and *Cyp11a1* were significantly increased in the MGCs cultured without oocytes and E2 (*p* < 0.05). On the other hand, oocytes completely suppressed the expressions of these transcripts in MGCs, consistent with the well-known function of oocytes in suppressing MGC luteinization^5,20^. Even in the presence of oocytes, FOXL2 protein and *Foxl2* mRNA levels were not maintained unless the MGCs were co-treated with E2 (Fig. 3A,B).

These results suggest that FOXL2 expression in MGCs requires stimulation with oocyte-derived signals and E2, at least under the present culture condition.

### BMP15 and GDF9 signals are required for maintaining FOXL2 expression by MGCs

**Figure 4:**
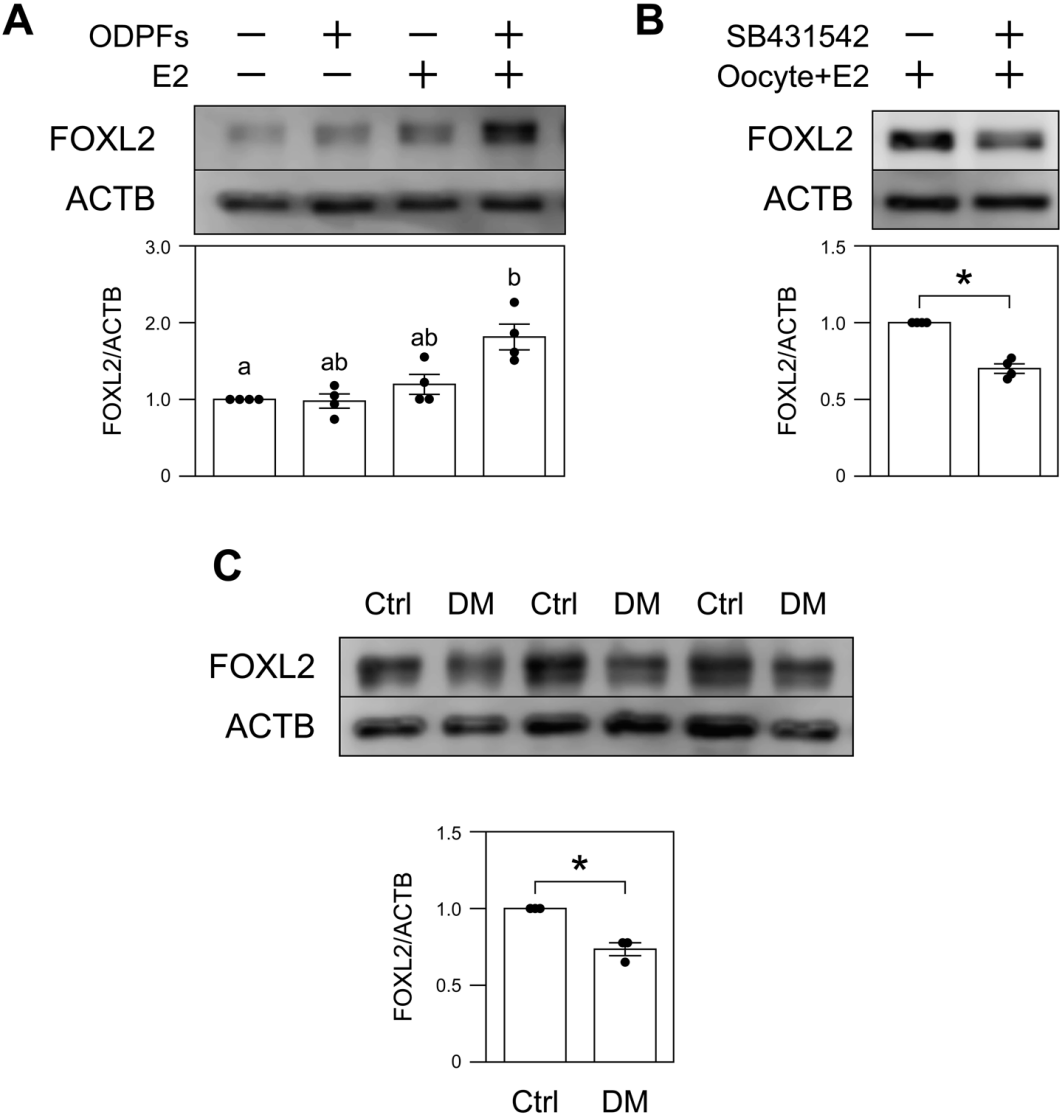
Effects of BMP15 and GDF9 signals on FOXL2 expression in MGCs. (**A**) MGCs were cultured with or without recombinant BMP15/GDF9 (ODPFs) and E2 for 24 h (n = 4). (**B**) MGCs cultured with both oocytes and E2 were treated with or without an inhibitor of ALK5, SB431542 for 24 h (n = 4). (**C**) Comparison of the expression levels of FOXL2 protein between MGCs of *Bmp15*^−/−^/*Gdf9*^+/−^ mice (DM) and those of littermate control *Bmp15*^+/−^/*Gdf9*^+/−^ mice (Ctrl) (n = 3 each). The expression of FOXL2 protein was examined by western blotting (upper panel) and band intensities were quantified (lower panel). Asterisks or different letters (a and b) denote significant differences (*p* < 0.05).

BMP15 and GDF9 are oocyte-derived paracrine factors (ODPFs) well known to play critical roles in the regulation of granulosa cell development and function^21^. To test the possibility that BMP15 and/or GDF9 regulate FOXL2 expression in MGCs in cooperation with E2, MGCs were co-treated with recombinant BMP15 and GDF9 together with E2, and then the FOXL2 levels were examined (Fig. 4A). The results showed that the level of FOXL2 expression in MGCs was significantly promoted when the cells were cultured with both of the ODPFs and E2.

The signal of GDF9 in granulosa cells is mediated by transforming growth factor, beta receptor I (TGFBR1, also known as ALK5). Next, to test whether endogenous GDF9 secreted from oocytes is indeed required for elevated FOXL2 expression in MGCs, MGCs co-cultured with oocytes and E2 were treated with or without an ALK5 inhibitor, SB431542, and the levels of FOXL2 were determined. While co-treatment of oocytes and E2 maintained FOXL2 expression in MGCs, the levels of FOXL2 were significantly decreased when the MGCs were treated with SB431542 (Fig. 4B). This suggests that oocyte-derived GDF9 signals are required for maintaining elevated FOXL2 expression in MGCs in vitro.

Since the ovaries of *Gdf9*-deficient mice exhibit arrest of folliculogenesis at the primary stage, there are no in vivo models for *Gdf9*-deficient MGCs. As an alternative, *Bmp15*^*-/-*^*/Gdf9*^+*/-*^ (double mutant; DM) mice are a well-accepted experimental model for the deficiency of oocyte-derived signals^3,21^. Therefore, to examine the requirement of ODPFs for FOXL2 expression in MGCs in vivo, FOXL2 expression in MGCs of DM mice were examined (Fig. 4C; DM mice are denoted as DM). The results showed that FOXL2 levels were slightly but significantly lower in MGCs of DM mice compared with those of littermate controls (*Bmp15*^+/−^/*Gdf9*^+/−^ mice; designated as Ctrl mice).

These results strongly suggest that ODPFs—most importantly GDF9—are required for the maintenance of FOXL2 expression in MGCs in vivo*.*

### Inhibition of estrogen signaling promotes precocious follicular development and elevation of FOXL2 expression in vivo

**Figure 5:**
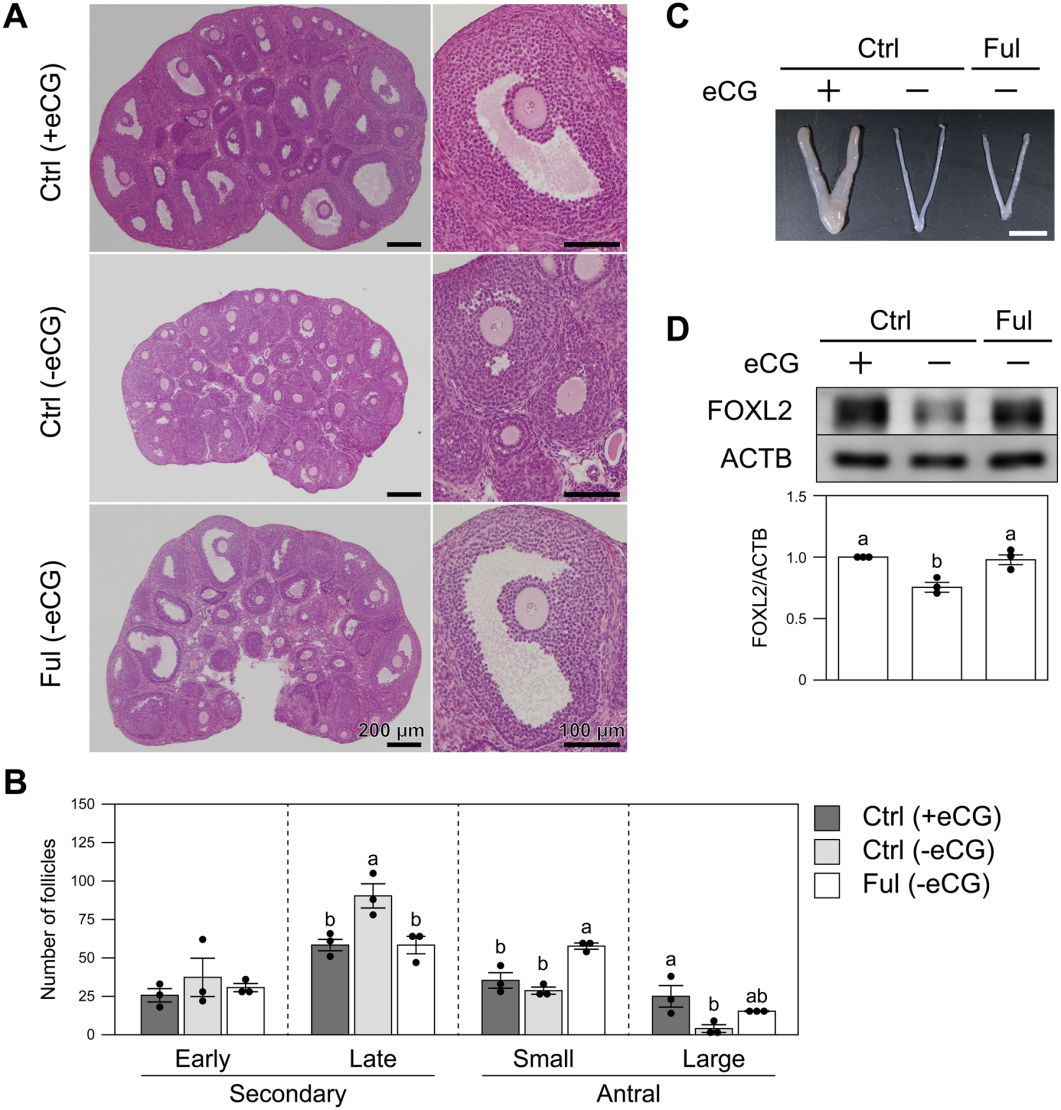
Effects of inhibition of estrogen signaling in vivo*.* (**A**) Ovaries of fulvestrant (Ful) or vehicle (Ctrl)-injected mice with or without eCG treatment were sectioned and stained with H & E. Scale bar, 200 and 100 μm for left and right panels, respectively. A small or a large antral follicle in each section is shown in the right panels. (**B**) The number of secondary and antral follicles of each ovary was counted (n = 3). (**C**) Morphology of a uterus isolated from a fulvestrant (Ful) or vehicle (Ctrl)-injected mouse. Scale bar, 5 mm. Ctrl (+ eCG), n = 3; Ctrl (-eCG), n = 6; Ful (-eCG), n = 8. (**D**) MGCs were isolated from fulvestrant (Ful) or vehicle (Ctrl)-injected mice and FOXL2 expression was examined by western blotting (upper panel). Band intensities were quantified (lower panel) (n = 3). Different letters (a and b) denote significant differences (*p* < 0.05).

Finally, since estrogen signaling promoted FOXL2 expression in concert with oocyte-derived signals in vitro (Fig. 3), we examined the effect of the inhibition of estrogen signaling in vivo using an estrogen receptor antagonist, fulvestrant (Fig. 5). Mice were injected every three days with either fulvestrant or vehicle from PD15 to PD21, and the ovarian histology and FOXL2 levels were examined at PD22. As shown in the middle panel of Fig. 5A, ovaries of equine chorionic gonadotropin (eCG)-unprimed control mice that had been injected with vehicle contained many secondary follicles and some early antral follicles. Ovaries of eCG-unprimed control mice contained significantly more large secondary follicles than those of other treatment groups (Fig. 5B). On the other hand, ovaries of fulvestrant-injected mice, although they were not primed with eCG, contained significantly more small antral follicles than other ovaries. The number of large antral follicles in fulvestrant-injected mice tended to be higher than eCG-unprimed control mice, and were comparable to those in eCG-primed controls (Fig. 5B). The uterus of fulvestrant-injected mice was much thinner than that of the eCG-primed controls, and was comparable in appearance to that of eCG-unprimed controls (Fig. 5C). This phenotype of fulvestrant-injected mice resembles the precocious maturation of ovarian follicles in the prepubertal ESR1/2 double-knockout mice^8^, strongly suggesting that estrogen signaling was correctly inhibited in the present experimental model. Unexpectedly, however, fulvestrant injection significantly enhanced the FOXL2 protein level in MGCs compared with the level in eCG-unprimed controls. The FOXL2 level in MGCs of the fulvestrant-injected mice was comparable to that in eCG-primed controls (Fig. 5D). Inhibition of estrogen signaling induced precocious follicular development in vivo, and this precocious follicular maturation may be the reason for the elevated FOXL2 expression in MGCs of the fulvestrant-injected mice (please see Discussion).

## Discussion

The various cells making up the ovarian follicles, including oocytes, cumulus cells, and MGCs, clearly do not develop on their own, but rather require complex communication with each other. In fact, it is well accepted that the bi-directional communication between oocytes and cumulus cells is critical for the normal development of both cell types^22^. MGCs contribute to this oocyte–cumulus cell communication through the production of estrogen as oocyte-derived signals and estrogen cooperatively regulate the development and function of cumulus cells^9,10,15,22^. On the other hand, the mechanism by which oocytes or cumulus cells affect the development of MGCs has not been well understood. Herein, we showed that the expression of FOXL2, a critical regulator of MGC development, was maintained in MGCs through the cooperative interaction of oocytes and estrogen. This would explain, at least in part, how oocytes affect MGC development. Therefore, oocytes seem to play a critical role in regulating the development and function of both MGCs and cumulus cells. The well-developed MGCs probably produce more estrogen, and estrogen indirectly promotes the development of oocytes by promoting cumulus cell development. Importantly, the estrogen production by MGCs is promoted by FSH^23–25^; this is a possible mechanism by which FSH promotes the development of cumulus cells and oocytes. Of course, while these communications may explain some portion of the complex mechanism governing follicle development, they probably do not account for all of it. Further studies testing these possibilities are warranted.

The present results suggest that BMP15 and GDF9 are essential for the expression of FOXL2 in MGCs. The synergistic interaction of BMP15 and GDF9 has been reported in many studies. For example, our previous study showed that FOXL2 expression in cumulus cells is suppressed by BMP15 and GDF9^14^. The mechanism of the synergism has been attributed, at least in part, to a heterodimerization of BMP15 and GDF9^26^. Determining whether the BMP15:GDF9 heterodimer, designated as “cumulin”^27^, is involved in the regulation of FOXL2 expression in MGCs will require further investigation. In addition, in our present experiments, while FOXL2 was barely detectable in MGCs after 72 h of culture (without oocytes or estrogen), MGCs isolated from DM mice exhibited only a slight reduction in the FOXL2 level compared to those of litter-mate controls. Likewise, the inhibition of ALK5 signaling in MGCs by SB431542 only partially suppressed the oocyte/estrogen maintenance of FOXL2 levels in vitro. While GDF9 homodimer produced in DM mice may account for the FOXL2 expression in the mutant mice to some extent, it is likely that other oocyte factors also participate in the maintenance of FOXL2 expression in MGCs in vivo. For instance, oocytes produce several other growth factors, such as BMP6, TGFB2 and fibroblast growth factors (FGFs), which act in concert to regulate the development and function of granulosa cell populations^28–30^. In addition to these oocyte factors and estrogen, it is likely that well-developed antral follicles contain some unknown factors, which promote FOXL2 expression by MGCs in vivo. In fact, our previous report showed that *Foxl2* levels of primed MGCs were significantly higher than those of unprimed MGCs^14^. In the present study, precocious follicular development was induced by inhibition of estrogen signaling in vivo and resulted in promotion of FOXL2 expression in MGCs. The fulvestrant treatment might suppress the estrogen negative feedback on the pituitary gland, which might result in an increase in the endogenous FSH and, therefore, a precocious development of antral follicles. Thus, the precocious maturation of ovarian follicles induced by estrogen inhibition resulted in up-regulation of the FOXL2-promoting activity; and it may mask the direct effects of inhibiting estrogen signaling on FOXL2 expression in MGCs. Alternatively, other estrogen receptor, such as GPR30, which is not suppressed but rather activated by fulvestrant treatment^31^, may mediate E2 effects on FOXL2 expression by MGCs. These possibilities need to be tested in future analyses; however, the results in this study strongly suggest that signals of BMP15/GDF9 and estrogen facilitate FOXL2 expression in MGCs.

In a previous study, we reported that *Esr2* expression is decreased in our culture system^32^. In the same study, we also showed that *Esr2* expression in MGCs was not promoted by stimulation with FSH or ODPFs. However, the cultured MGCs may maintain some level of ESR2 expression sufficient to allow their response to estrogen, since these cells did respond to E2 in the present study. If one could maintain ESR2 expression at higher levels during the culture, MGCs would have more responsiveness to E2, and therefore might exhibit a more drastic elevation in FOXL2 expression.

How do oocyte and estrogen signals promote FOXL2 expression in MGCs? In contrast to the process of sex determination, the mechanism by which FOXL2 expression is maintained in granulosa cells of antral follicles has not been well studied. During embryonic development, WNT4 signaling plays a critical role in promoting FOXL2 expression in developing gonads, thereby leading to female development. Therefore, WNT4 signaling may also promote FOXL2 expression in MGCs in antral follicles. In fact, conditional deletion of *Ctnnb1* (which encodes β-catenin) in early antral follicles decreases—whereas forced expression of dominant stable CTNNB1 promotes—the expression of *Cyp19a1* mRNA, one of the transcriptional targets of FOXL2 in granulosa cells in mice in vivo^33^. Interestingly, SMADs and WNT/β-catenin pathway components can physically interact to mutually regulate one another’s activity. For example, SMAD3 plays a critical role in shuttling β-catenin into the nucleus in human mesenchymal stem cells^34^. Moreover, regulators of the SMAD signaling, such as SMURF1/2 and SMAD7, can also mediate signal crosstalk with the WNT pathway^35–37^. In addition, WNT4 mediates the effects of estrogen receptor signaling in an invasive lobular carcinoma cell line^38^. Therefore, it may be possible that oocytes and estrogen promote *Foxl2* expression in MGCs by interacting with WNT/β-catenin signaling. Alternatively, BMP15/GDF9 signaling may directly promote *Foxl2* expression in MGCs, as several potential SMAD responsive elements^39^ are found in the regulatory region of the *Foxl2* gene in mice (not shown).

The present results showed FOXL2 expression in MGCs is promoted by oocytes, whereas our previous study showed FOXL2 expression in cumulus cells is suppressed by oocytes^14^. Therefore, signals of oocytes have contrast effects on FOXL2 expression between these cell types. While cumulus cells and MGCs originated from the same cell type, i.e., granulosa cells of preantral follicles, they are indeed developed into different cell types. In fact, we previously reported that many transcriptional regulators, such as transcription factors and epigenetic modifiers, are differentially expressed between cumulus cells and MGCs^14^. These differences in expression of transcriptional regulators likely account for the functional diversification of these cell types and, therefore, for the differential responses between these cell types to oocyte signals.

Conditional deletion of *Foxl2* in the adult mouse ovary results in up-regulation of SOX9 in granulosa cells mainly located in the outer portion of early antral follicles, i.e., putative future MGCs, and ultimately the trans-differentiation of the granulosa cells into Sertoli-like cells^13^. This suggests that the maintenance of the granulosa cell phenotype in vivo requires an active mechanism which involves the suppression of SOX9 by FOXL2^13^. Therefore, we expected that the FOXL2-decreased MGCs in our culture system would exhibit elevated SOX9 expression and acquire the characteristics of Sertoli cells. In fact, the level of *Sox9* transcripts was significantly increased in MGCs maintained without oocytes or estrogen. However, the *Sox9* level was notably lower than that in testicular cells, and SOX9 protein expression was barely observed in the cultured MGCs. While further studies need to be conducted, these results suggest that the decrease in the FOXL2 level may not be sufficient to completely de-repress *Sox9* expression and to induce the trans-differentiation of MGCs into Sertoli-like cells, at least under the present culture condition*.*

There are several possible explanations for the increase in *Sox9* mRNA without detectable SOX9 protein expression in the present experiment. SOX9 protein levels are regulated by multiple mechanisms, including post-transcriptional regulation of SOX9 mRNA and post-translational modifications. In fact, several micro RNAs (miRNAs) are reported to target *SOX9/Sox9* mRNAs in mammalian species, including humans and mice^40^. The translation of *Sox9* mRNA is controlled by the mechanistic/mammalian target of rapamycin (mTOR) signaling through the inhibition of the Eukaryotic translation initiation factor 4E-binding proteins in mesenchymal stem cells in mice^41^. Moreover, long non-coding RNAs are also involved in the regulation of SOX9 protein levels in chondrocyte differentiation and in lung adenocarcinoma^42,43^. Therefore, similar mechanisms may be present and prevent an increase in SOX9 protein levels in murine MGCs. Since the upregulation of SOX9 protein would promote trans-differentiation of granulosa cells into Sertori-like cells in vivo^13^, such mechanisms would be critical for maintaining the "integrity" of granulosa cells, and therefore, the maintenance of ovarian function.

The causative effects of the FOXL2 misregulations have been identified in several ovarian diseases, including primary ovarian insufficiency (POI)^44^ and granulosa cell tumor^45^. While the mechanism by which the FOXL2 misregulations induce such ovarian diseases has been actively investigated, the mechanisms underlying the regulation of FOXL2 expression are not known. Therefore, the present finding that oocyte-derived signals and estrogen are the critical upstream regulators of FOXL2 expression in MGCs is likely to elicit novel insights regarding the complex mechanism governing ovarian pathogenesis. In fact, a significant association has been reported between some *BMP15* variants and POI in humans^46^. The present results imply that such ovarian defects associated with deficiencies in ODPFs or estrogen signaling may be attributable to attenuated FOXL2 expression. Further investigation of ovarian defects focusing on FOXL2 and its upstream regulators, i.e., oocytes and estrogen, will provide new insights into the pathogenetic mechanisms and their therapeutic targets.

## Materials and methods

### Mice

(C57BL/6 N x DBA/2) F1 (BDF1) mice were purchased from Sankyo Lab Service (Tokyo, Japan) or produced and raised in the research colony of the investigators at the University of Tokyo. In some experiments, *Bmp15*^−/−^/*Gdf9*^+/−^ mice (DM mice) on a C57BL/6 N/CD-1 mixed background were used. The mice were housed under a 12-h light/12-h dark schedule and provided with food and water ad libitum. At the end of the experiment, all mice were euthanized by cervical dislocation. All the animal experiments were conducted in accordance with the institutional guidelines and approved by the institutional Animal Care and Use Committees at the University of Tokyo. The experiments were performed and reported in accordance with ARRIVE guidelines.

### Culture medium

The basic culture medium was bicarbonate buffered MEMα (Minimum Essential Medium α; Thermo Fisher Scientific, Gaithersburg, MD, USA) supplemented with 75 μg/ml of penicillin G, 50 μg/ml of streptomycin sulfate, and 3 mg/ml of bovine serum albumin (BSA; Sigma-Aldrich, St. Louis, MO, USA). When investigating the effect of oocytes on MGCs, the basic culture medium supplemented with 10 nM of the phosphodiesterase inhibitor, milrinone (Sigma-Aldrich), was used to prevent spontaneous maturation of oocytes. In some experiments, the basic culture medium was supplemented with 10^–7^ M of 17β-estradiol (Sigma-Aldrich), recombinant human BMP15 (50 ng/ml; R&D Systems, Minneapolis, MN, USA), recombinant mouse GDF9 (50 ng/ml; R&D Systems), and/or an inhibitor of ALK5, SB431542 (10 μM; FUJIFILM Wako Pure Chemical Corporation, Osaka, Japan).

### Isolation and culture of cells

MGCs were isolated from 3-week-old BDF1 mice and cultured as previously reported^47^. Briefly, isolated MGCs were centrifuged and re-suspended in the basic culture medium, and 4 × 10^4^ cells were cultured in individual wells of a 96-well plate (IWAKI, Tokyo, Japan) pre-coated with ECL cell attachment matrix (Merck Millipore, Burlington, MA, USA). After 24 h of culture, cells were washed and cultured for an additional period in the basic culture medium with or without supplements. In some experiment, MGCs were co-cultured with oocytes isolated from 3-week-old BDF1 mice at a concentration of two oocytes per microliter. Three to five mice were used to isolate MGCs in each culture experiment. The culture periods and supplements are described in the figure legend. All cultures were maintained at 37 °C in 5% CO_2_. Culture experiments were repeated at least three times and the samples were used in the following analyses individually.

In some experiments, MGCs were isolated from 3-week-old DM and littermate control (*Bmp15*^+/−^/*Gdf9*^+/−^) mice that were injected with 6 IU of equine chorionic gonadotropin (eCG; ASKA Pharmaceutical, Tokyo, Japan) 45–46 h earlier and used to detect FOXL2 levels by western blotting. Three DM mice and three control mice were used each and the results of western blotting analysis for all samples were shown in the figure (Fig. 4C, upper panel).

### Fulvestrant treatment

Fulvestrant (Tokyo Chemical Industry, Tokyo, Japan), an estrogen receptor antagonist, was used to inhibit estrogen signaling in vivo. Fulvestrant was prepared in 10% DMSO in corn oil (FUJIFILM Wako Pure Chemical Corporation). Mice received s.c. injections of fulvestrant (1 mg/mouse) or vehicle every three days from postnatal day (PD) 15 to PD21. Mice received i.p. injection of the three types of mixed anesthetic agents (0.075 mg/ml medetomidine, 0.4 mg/ml midazolam, and 0.5 mg/ml butorphanol)^48^ before every fulvestrant or vehicle injections, and then received i.p. injection of 0.075 mg/ml atipamezole to facilitate recovery. Some mice were i.p. injected with eCG at PD20. To minimize the potential confounding factors that would affect the results, all mice were numbered and co-housed in the same cage during the course of each experimental repeat. Ovaries and granulosa cells were isolated at PD22 and used for histological analyses and western blotting, respectively. The experiments were repeated at least three times. The numbers of mice per experimental group are described in the figure legend. Sample size was not predetermined and all mice were included in the analyses.

### Histological analysis

Ovaries were fixed with Bouin’s solution and embedded in paraffin (Pathoprep568; FUJIFILM Wako Pure Chemical Corporation). Ovarian blocks were serially sectioned (thickness, 6 μm) and stained with H&E in the usual manner.

The numbers of follicles at each developmental stage were estimated by counting follicles in every third section where the germinal vesicle of the oocyte was observed^49^. The criteria for classifying follicular stages were as follows: early or late secondary follicle, more than one or two layers, respectively, of granulosa cells surrounding the oocyte without antrum formation; small antral follicle, small antrum is in the process of forming; and large antral follicle, a single antral space separating the MGCs from the cumulus cells surrounding the oocyte.

### Real-time quantitative reverse transcription PCR (qPCR)

qPCR was conducted as previously reported^9^. Briefly, total RNA was extracted from cells using a ReliaPrep RNA Cell Miniprep System (PROMEGA, Tokyo, Japan) and reverse-transcribed using a ReverTra Ace qPCR RT Master Mix with gDNA Remover kit (TOYOBO, Osaka, Japan) in accordance with the manufacturer’s protocols. The qPCR was performed using THUNDER BIRD qPCR Mix (TOYOBO) and an ABI StepOne Real-time PCR System (Applied Biosystems, Foster City, CA, USA). The transcript levels were standardized to the levels of a housekeeping gene, ribosomal protein L19 (*Rpl19*), by the 2-^ΔΔCt^ method^50^. All the PCR reactions were conducted in duplicate. A melt curve analysis was performed at the end of the amplification process to confirm the specificity of the PCR products. In addition, only one product of the estimated size was identified by agarose gel electrophoresis for each set of primers. The PCR primers are shown in Table 1. *Cyp11a1* and *Star* primers were reported previously^49^. All the experiments were conducted more than three times independently.

**Table 1:** Primer sets used for qPCR.

Refseq Acc. No	Gene symbol	Forward primer sequence	Reverse primer sequence
NM_011448	*Sox9*	GAGGAAGTCGGTGAAGAACG	CTGAGATTGCCCAGAGTGCT
NM_012020	*Foxl2*	GAGCGGTCCCCCACCCCTATC	CGGAGGCGACAAAGCGGAGT
NM_001301375	*Fst*	CTCCTCAAGGCCAGATGCAA	TGGAGCTGCCTGGACAAAAA
NM_007810	*Cyp19a1*	AGCAGCAATCCTGAAGGAGA	AGCCGTCAATTACGTCATCC
NM_009078	*Rpl19*	CCGCTGCGGGAAAAAGAAG	CAGCCCATCCTTGATCAGCTT

### Western blotting

Western blotting was performed as previously reported^14^. The primary antibodies used were anti-FOXL2 antibody (1:1000; 1 μg/ml; Cat. No. F0805; RRID: AB_1078904; Sigma-Aldrich), anti-ACTB antibody (1:1000; 0.09 μg/ml; Cat. No. GTX109639; RRID: AB_1949572; GeneTex, Irvine, CA, USA), or anti-SOX9 antibody (1:1000; 1 μg/ml; Cat. No. AB5535; RRID: AB_2239761; Merck Millipore). The secondary antibody used was peroxidase conjugated anti-rabbit IgG antibody (1:3000; 0.3 μg/ml; Cat. No. AP132P; RRID: AB_90264; Merck Millipore). Signals were visualized using an Immunostar LD kit (FUJIFILM Wako Pure Chemical Corporation) and a C-DiGit Blot Scanner (LI-COR, Lincoln, NE, USA). The protein levels were quantified using ImageJ software^51^ (version 1.53 k; NIH, Bethesda, MD, USA).

### Statistical analyses

Statistical analyses were performed using JMP Pro Version 15 statistical analysis software (SAS Institute, Cary, NC, USA). Student's *t*-test and the Tukey–Kramer test were used for the pairwise and the multiple comparisons, respectively. Values of *p* < 0.05 were considered to indicate statistical significance. All values were shown as mean ± SEM.

## Supplementary Information

Supplementary Information.

## Data Availability

The data underlying this article will be shared on reasonable request to the corresponding author.
