# Supplementary material for: Cooperative effects of oocytes and estrogen on the forkhead box L2 expression in mural granulosa cells in mice
Source: Sci Rep. 2022 Nov 23;12:20158. doi: 10.1038/s41598-022-24680-x (PMC9691737; doi:10.1038/s41598-022-24680-x)
Supplement: Supplementary file 1 — Supplementary Information. [file 41598_2022_24680_MOESM1_ESM.pdf]

Supplementary Information

Cooperative effects of oocytes and estrogen on the Forkhead box L2 expression in mural granulosa cells in mice

Haruka Ito[1], Chihiro Emori[1, 2], Mei Kobayashi[1], Natsumi Maruyama[1], Wataru Fujii[1], Kunihiko Naito[1], Koji Sugiura[1, *]

1 Laboratory of Applied Genetics, Department of Animal Resource Sciences, Graduate School of Agricultural and Life Sciences, The University of Tokyo, Tokyo, Japan

2 Present address: Department of Experimental Genome Research, Research Institute for Microbial Diseases, Osaka University, Suita, Osaka, Japan

*Correspondence and requests for materials should be addressed to K.S. (e-mail: aks@g.ecc.u-tokyo.ac.jp)

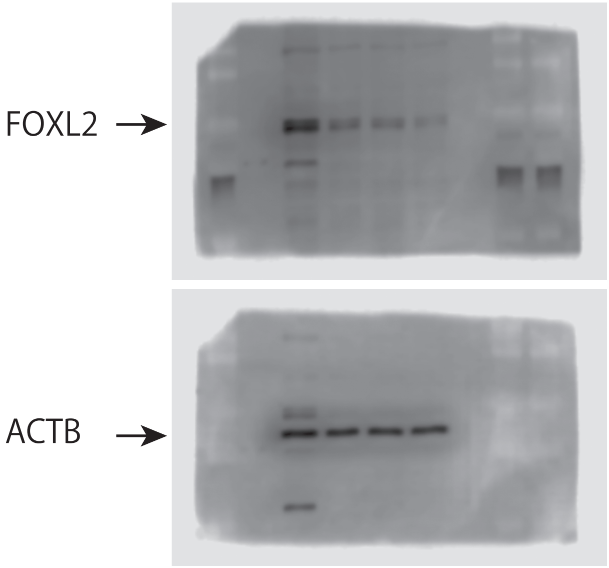

Supplementary Figure S1. Original scanned image of western blot analysis shown in Figure 1A.

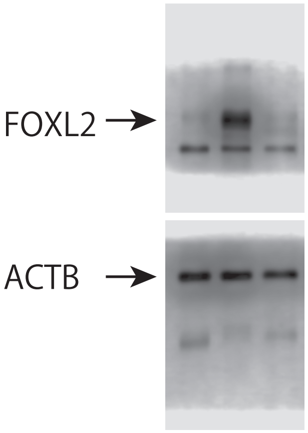

Supplementary Figure S2. Original scanned image of western blot analysis shown in Figure 2D.

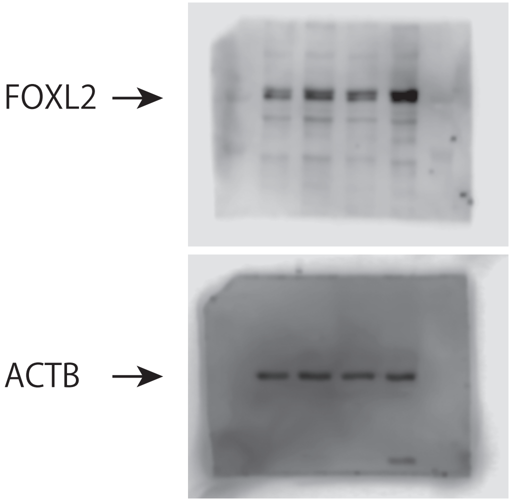

Supplementary Figure S3. Original scanned image of western blot analysis shown in Figure 3A.

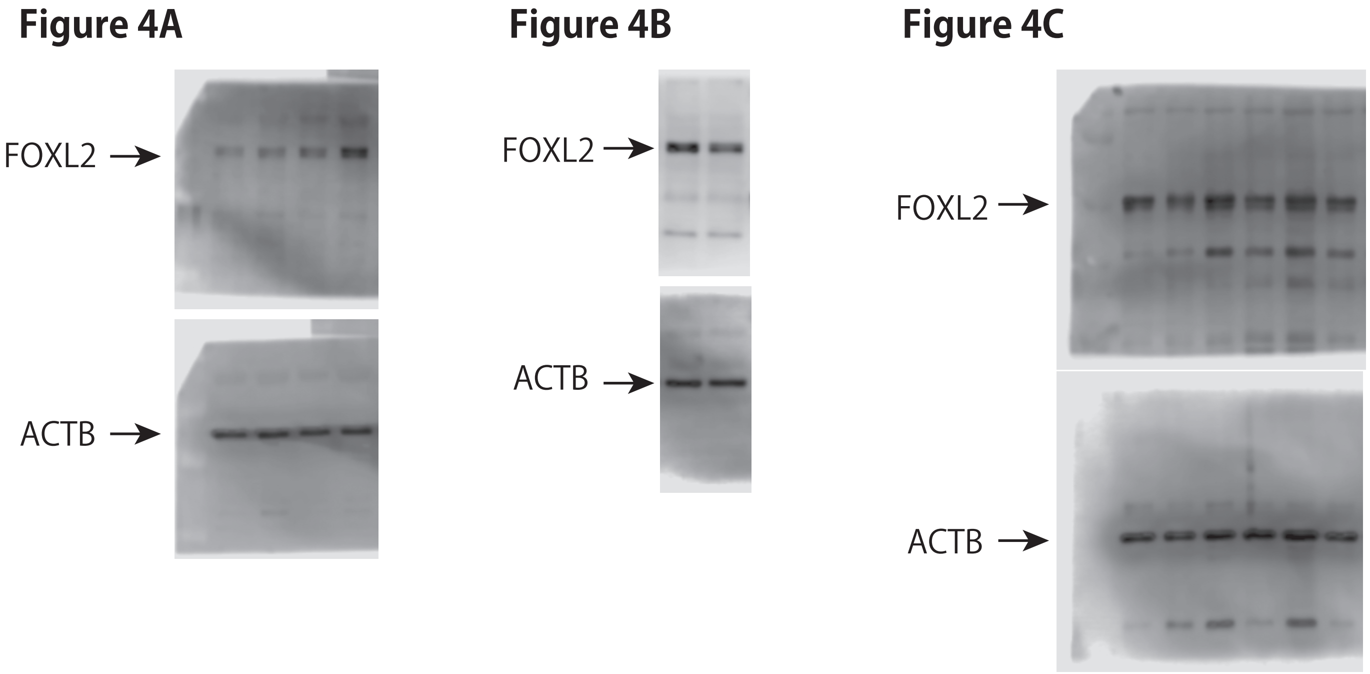

Supplementary Figure S4. Original scanned image of western blot analysis shown in Figure 4.

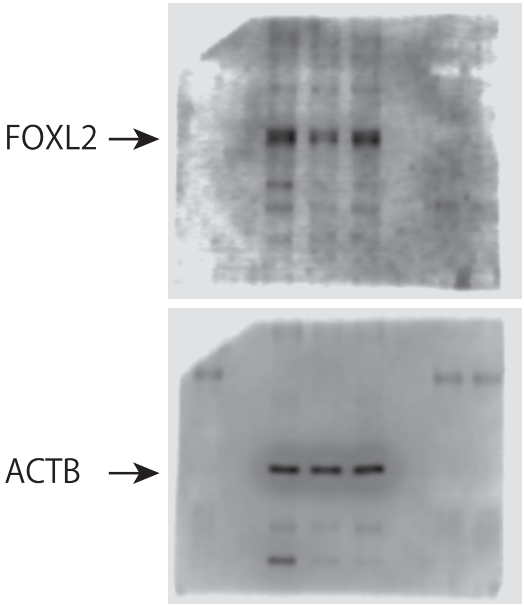

Supplementary Figure S5. Original scanned image of western blot analysis shown in Figure 5.
